# Views on fall prevention among multi-disciplinary healthcare workers

**DOI:** 10.6026/973206300211827

**Published:** 2025-07-31

**Authors:** Geetha D., Vijayasamundeeswari P., Angel Preethi M.

**Affiliations:** 1 Sri Ramachandra Faculty of Nursing, SRIHER (DU), Porur, Chennai 116, India

**Keywords:** Fall, healthcare professionals, inpatient, multidisciplinary, prevention

## Abstract

Falls are a common and inevitable occurrence in healthcare settings. Nurses are often questioned about these incidents and are responsible for reporting them. When the multidisciplinary team is actively involved, it becomes easier to reduce fall rates in healthcare settings. Therefore, it is of interest to assess healthcare professionals' perceptions of fall prevention and to develop an informative video on the subject. A descriptive design was adopted; the target population was multidisciplinary Health Professionals working at teaching hospitals, South India. The stratified sampling technique was used to recruit the samples in the study, the calculated ratio was 0.414. Results show that, consistent monitoring, education and evaluation of fall prevention practices are essential for reducing the occurrence of falls among vulnerable individuals in hospitals.

## Background:

Patient safety has emerged as a pressing global concern, requiring competence across diverse domains such as human factors and systems engineering. International health authorities have increasingly emphasized system-level strategies to address safety challenges, recognizing that well-designed healthcare systems are crucial to delivering safe and effective care [1]. Among the various patient safety issues, falls represent a significant public health burden worldwide. Each year, approximately 684,000 individuals die from falls, ranking them as the second most common cause of accidental injury-related deaths after road accidents [2]. Studies indicate that falls in hospital settings occur at a rate of 3-5 per 1000 patient days. The Agency for Healthcare Research and Quality (AHRQ) estimates that annually, between 700,000 and 1 million patients experience a fall while hospitalize [3]. In NHS hospitals, falls constitute the most frequently reported safety incidents, with physical injuries resulting in 30-50% of cases and fractures occurring in 1-3% [4]. Falls not only lead to physical harm but also have psychological repercussions, such as reduced confidence, delayed rehabilitation and extended hospital stays. Healthcare providers play a pivotal role in patient safety by assessing and managing fall risks. Nurses, in particular, are actively engaged in fall prevention as part of routine patient care-conducting regular fall assessments, identifying at-risk patients and marking beds with fall alerts. The consequences of falls range from minor skin injuries to severe outcomes like head trauma, bone fractures and even mortality [5]. In a study conducted at a large urban academic hospital, involving 1,300 beds over 13 weeks, 183 inpatient falls were recorded. The average age of those who fell was 63.4 years. Most falls occurred unassisted (79%); within patient rooms (85%), predominantly during evening or night shifts (59%) and often while the patient was walking (19%) [6]. Collaborative efforts between patients and healthcare teams have shown promise in reducing fall incidents and related injuries [7]. Therefore, it is of interest to explore the perspectives of multidisciplinary healthcare professionals regarding fall prevention practices in selected hospitals.

## Methodology:

This was a quantitative study examining perspective on fall prevention among multidisciplinary health professionals. After approval by the institutional review boards and the ethical committee the perspective questionnaire was prepared. The study adopted stratified sampling techniques where the health care professionals were stratified into subgroups to achieve the sample size. Demographic data that were collected included years of experience, educational level, unit worked and previous knowledge on fall prevention in hospital. Fall Prevention Knowledge was measured by a questionnaire developed by the investigator based on the literature review. The Fall Prevention comprehensive perspective Questionnaire consisted of the fall risk assessment, risk factors, complication after fall and post fall measures. The questionnaire consisted of 35 items which was applicable for the registered nurses and 27 items for the other healthcare personnel. Using two different links for the health care professionals, the data was collected. The content validity of the questionnaire was confirmed by experts in the field of quality improvement, fall nurse and academician. The results showed that the test-retest reliability of the questionnaire was r =0.98. Fall Preventive competency was measured by the questionnaire developed by the investigator based on the literature review [8]. The Fall Preventive competency perspective Questionnaire consisted of the behaviour adhered to prevent fall in hospital [9]. The tool consisted of 18 items rated on 5-point likert scale for both the registered nurses and other healthcare professionals circulated through Google form. The results showed that the test-retest reliability of the questionnaire was r =0.90.The comprehensive and competency perspective questionnaires were circulated using the Google form. Each participant was given 15-20 minutes under the guidance of the investigator. The data was collected for a four-week period of time. The video was developed in order to enhance the fall prevention activities among patients [10].

## Results & Discussion:

A total of 425 samples were collected out which five found to be incomplete hence, the data analysis was done with 420 samples. Out of 420 registered nurses, 228 (86%) were female, 182 (68%) were between the ages of 21 and 30 and 112 (42%) had 2 to 5 years of experience. In addition, 16 (61%) had a bachelor's degree, 233 (89%) had knowledge of fall prevention in hospital settings and 31 (11%) did not. The data came from health professionals, mostly women (58%). with the remaining 65% (42%) being male, revealed the following: 114 (73%) were between the ages of 21 and 30. In terms of experience, 89 (57%) were between 2 and 5 years old and 60 (38%) were under one year of age. The mean and standard deviation of the level of fall prevention knowledge of hospital-registered nurses were 22,958, with a standard deviation of 4,068. In contrast, for other healthcare professionals, the mean score was 17,173 with a standard deviation of 4.209 and the standard deviation and mean of the practice of hospital-certified nurses in fall prevention falls, showing a mean score of 69.556 with a standard deviation of 4,209. For other healthcare professionals, the mean score was 61,442 with a standard deviation of 9.314. Table 1 (see PDF) Knowledge mean score of registered nurses was 22.958 with a standard deviation of 4.068. On the other hand, for other health care professionals, the mean score is 17.173 with a standard deviation of 4.209. Table 2 (see PDF) shows 157(59%) registered nurses, 13(13%) residents/duty doctors, 5(20%) allied healthcare professionals, 3(14%) other paramedical hospital staff and 4 (31%) nursing assistants demonstrated satisfactory Perspective. Table 3 (see PDF) presents the distribution of Competency levels among healthcare professionals 139 (53%) registered nurses, 81 (84%) resident/duty doctors, 18 (7%) allied health professionals, 15 (71%) other paramedical healthcare professionals and 69 (9%) nursing assistants demonstrated moderately satisfactory Competency. In the category of satisfactory Competency, 122 (46%) were registered nurses, 15 (15%) were resident/duty doctors, 5(20%) were allied health professionals, 6 (29%) were other paramedical healthcare professionals and 4 (31%) were nursing assistants.

The present study revealed that fall incidents were observed by the study participants across different age groups, with 7% occurring among individuals aged 19 to 35 years and another 7% in those aged 56 years and above. When analyzed by gender, 10% of the falls involved female patients, while a higher proportion (19%) involved male patients. The most common locations for falls were restrooms (14.4%), followed by bedsides (13.6%) and corridors (2%) [11]. Table 4 (see PDF) shows the method of selecting the samples from population by stratified method. The overall Comprehensive mean score for the registered nurses on fall prevention in hospital was 22.958 (out of 35). The level of Comprehensive perspective among registered nurses showed that 15 (6%) had inadequate, 92 (35%) had moderate and 157 (59%) had adequate perspective on fall prevention in hospital (Table 3 - see PDF). The overall mean score for other health care professionals was 17.173 (out of 27). An adequate Comprehensive perspective was evident among the other health care professionals was 17 (18%) in residents and duty doctors, 6 (24%) in allied health workers, 5(24%) in other paramedical workers and 3 (23%) in nursing assistants. The moderate perspective among other health care professionals was 67 (69%) of residents/duty doctors, 14 (56%) of allied health workers, 13 (62%) in other paramedical worker and 6 (46%) nursing assistants and an inadequate comprehensive perspective was prominent among 13 (13%) residents/duty doctors, 5 (20%) allied health workers, 3 (14%) other paramedical hospitals and in 4 (31%) nursing assistants. A similar study showed the knowledge, attitude and fall prevention strategies all received fairly satisfactory scores. In the correlation between the knowledge and attitudes for falls and fall prevention activities, knowledge and attitudes about falls (r = 0.45, p < 0.001), knowledge and fall prevention activities (r = 0.27, p < 0.002) and fall attitude and preventive activities (r = 0.42, p < 0.001) showed statistically significant positive correlation [12]. Another study results showed that 6% of participants in this research had adequate knowledge about falls, 57.2% had favourable attitudes towards falls and 38.3% had adequate awareness of fall risk factors. Years of nursing experience are statistically significantly correlated with fall knowledge level. Age, education, nursing experience and prior patient falls all demonstrated statistically significant relationships with the participant's attitude towards falling. The relationship between fall awareness, fall-proneness and understanding of risk factors for falls was extremely significant. The vast majority of participants voiced support for the need for fall prevention education [13]. The study showing that by the end of the second PDSA cycle, the fall rate had decreased by 28% from the starting point. There were 99 admissions in total in September and 34 (34.3%) of those patients were classified as having a high fall risk. The reported fall rate was 3.8 per 1000 patient days. A third PDSA cycle was scheduled for October due to an increase in the fall rate in September. A total of 101 patients were admitted in total in October and 35 of them (34.6%) were classified as having a high risk of falling. In October, there was no fall occurrence. By the time the project was complete, they had nearly 90 days without a fall. When compared to the baseline, the average fall rate over the QI period (July to December 2021) was 1.56 per 1000 patient days. The results show that with increased practice on fall prevention methods the rates of fall had decreased [14]. Hospitals employ various measures like regular check-ins, bed alarms and mobility aids to prevent falls [15], but these precautions cannot entirely eliminate the inherent unpredictability of such incidents. Nonetheless, healthcare facilities make dedicated efforts to establish safe environments and educate patients and their families about fall risks, empowering them to play an active role in preventing falls [16]. Most of the literature shows that nurses had adequate knowledge and practice on fall prevention. Other health care professionals had a good degree of knowledge on fall prevention. The present study's video aimed at preventing falls was developed to validate by a panel of six experts. The assessment of content validity index of 0.83 with a universal agreement score of 0.85. For the purpose of establishing a valid instrument, it is generally recommended that the Content Validity Index (CVI) exceeds 0.80. The findings surpassed this threshold, providing evidence that the content presented in the educational video met the criteria of acceptability. These experts proposed interventions and precautionary measures to mitigate risks for patients dealing with delirium or dementia. Additionally, the experts suggested a reduction in the duration of the video.

## Conclusion:

Most nurses demonstrated sufficient knowledge and moderately satisfactory practice regarding fall prevention, while other healthcare professionals showed moderately sufficient knowledge and practice. Consistent monitoring, education and evaluation of fall prevention practices are essential for reducing the occurrence of falls among vulnerable individuals in hospitals. Thus, the significant responsibility of healthcare professionals and nurses in ensuring patient safety is needed.

## Compliance with ethical standards Ethical statement:

The study was conducted after approval with reference of CSP/22/JUL/114/437 from the Institutional Ethics Committee. The participants were explained clearly about the purpose of the study on Fall prevention perspectives. The written informed consent was obtained from all the participants before conducting the study. Confidentiality of responses was assured and maintained throughout the study.

## Financial support and sponsorship:

This project was self-funded
